# Behavioral Activation as an ‘active ingredient’ of interventions addressing depression and anxiety among young people: a systematic review and evidence synthesis

**DOI:** 10.1186/s40359-021-00655-x

**Published:** 2021-10-07

**Authors:** Kanika Malik, Maliha Ibrahim, Adam Bernstein, Rahul Kodihalli Venkatesh, Tara Rai, Bruce Chorpita, Vikram Patel

**Affiliations:** 1 Sangath, New Delhi, India; 2 Jindal School of Psychology and Counselling, O.P. Jindal Global University, Sonepat, Haryana India; 3 PracticeWise, LLC, Satellite Beach, USA; 4 Department of Psychology, Ashoka University, Sonepat, Haryana India; 5 Department of Psychology, University of California, Los Angeles, USA; 6 Department of Global Health and Social Medicine, Harvard Medical School, Boston, USA; 7 Harvard TH Chan School of Public Health, Boston, USA

**Keywords:** Behavioral activation, Systematic review, Lived experience, Transdiagnostic, Youth mental health, Active ingredient

## Abstract

**Background:**

Psychological interventions such as behavioral activation (BA) that focus on overt behaviors rather than complex cognitive skills may be developmentally well-suited to address youth mental health problems. The current systematic review synthesized evidence on the characteristics, effectiveness and acceptability of behavioral activation (BA) to examine its role as a potential ‘active ingredient’ for alleviating depression and anxiety among young people aged 14 to 24 years.

**Methods:**

Evidence across the following sources were synthesized: (i) randomized control trials (RCT) evaluating interventions where BA has been used as a standalone intervention or as part of a multicomponent intervention, (ii) qualitative studies examining the acceptability of BA as an intervention or as a coping strategy among young people with lived experiences. Consultations with a youth advisory group (YAG) from India were used to draw inferences from existing evidence and identify future research priorities.

**Results:**

As part of the review, 23 RCTs were identified; three studies examined BA as a standalone intervention, and the remaining studies examined multicomponent intervention where BA was a constituent element. The intervention protocols varied in composition, with the number of intervention elements ranging between 5 to 18. There was promising but limited evidence in standalone interventions for thse effectiveness of BA for depression. The impact of BA in multicomponent interventions was difficult to evaluate in the absence of focal assessment of activation outcomes. Evidence from 37 additional qualitative studies of youth lived experience literature, corroborated by the YAG inputs, indicated that young people preferred using behavioral strategies similar to BA to cope with depression in their own life. Themes indicated that the activities that are important to an individual and their socio-contextual factors need to be considered in the planning and implementing BA intervention. Evidence for the use of BA in anxiety was limited across data sources.

**Conclusions:**

Overall, there was preliminary empirical evidence for the effectiveness and acceptability of BA for youth depression. Further research is needed to examine the components and mechanisms that contribute to its effectiveness as an active intervention ingredient for depression and anxiety.

**Supplementary Information:**

The online version contains supplementary material available at 10.1186/s40359-021-00655-x.

## Background

Depression and anxiety are common, often co-occur, and are associated with significant disability among young people [1, 2], indicating a need for conceptually integrated and resource-efficient interventions that can address symptoms of both conditions. Although Behavioral Activation (BA), was initially conceptualized as an ‘active ingredient’ of interventions for adult depression [3, 4], its evidence for effectiveness as a transdiagnostic technique, is gradually emerging [5]. Cost-effectiveness, ease of delivery by non-specialists, and cultural sensitivity adds to the scalability of BA, particularly in the low-middle income countries (LMICs), where there is a paucity of financial and trained human resources [6–8].

BA involves systematically increasing individuals’ engagement in pleasurable activities, and in recent years the repertoire of activities has been expanded to include personally meaningful and valued activities [9]. The increased engagement with these overt behaviors is intended to bring the individual into contact with positive reinforcements in their environment, leading to more adaptive alternatives to withdrawn or avoidant behaviors, which underpin functional impairment in depression and anxiety [3, 10, 11]. The intervention elements that have been used as part of BA have varied across studies, with certain elements such as activity scheduling and self-monitoring being consistently present, whereas other elements such as skill training, personal values, goal assessment, and functional analysis have been variably used across studies and often as secondary to the above two components [12].

Most of the existing evidence on the utility and effectiveness of BA comes from studies with adults [13, 14]; however, the emphasis on concrete, overt behavior rather than complex emotional or cognitive skills makes it developmentally well-suited for young people [15]. Two recent systematic reviews of randomized and non-randomized BA studies provided preliminary evidence for its effectiveness among children and adolescents (below age of 19 years) with depression, with limited evidence for its use in anxiety [16, 17]. However, the limited number of randomized control trials (RCTs) and small sample sizes were a limitation to the strength of evidence in these reviews. Moreover, young people’s perception about the acceptability of BA in managing anxiety and depression, was not examined in the existing reviews.

The current rapid review was undertaken to synthesize evidence from multiple data sources (i.e., randomized control trials and lived experience literature) on the potential role of BA as an active ingredient for interventions aimed at young people with, or at risk for, depression and anxiety. The specific objectives of the study were to examine the characteristics and effectiveness of interventions where BA has been used, and the acceptability of BA among young people with, or at risk for, depression and anxiety. This rapid review was conducted as part of the Wellcome Trust’s Active Ingredients commission set out to explore potential intervention ingredients that address anxiety and depression in young people aged 14–24 years [18]. The target age group in the current study overlaps but is broader than the age groups included in previous BA review studies [16, 17].

## Methods

### Information sources

The rapid review was carried over period of four months (June-September 2020). Rapid reviews are literature reviews conducted systematically within a limited time frame and with specific databases [19]. We addressed the study’s objectives by drawing insights from two separate but complementary systematic reviews. The first review (quantitative review) was addressed objectives related to the characteristics and effectiveness of BA in depression and anxiety. Here we systematically synthesized RCTs on BA, in particular RCT studies that evaluated standalone BA interventions and multicomponent interventions with BA elements. The second review (qualitative review) addressed the objective related to the acceptability of BA in young people. In this review, we synthesized the literature on the lived experience of BA, in particular youths' experiences of participating in interventions with BA, and habitual use of BA like coping strategies to alleviate depression, anxiety, or both. We reasoned that the closer the match between what youth habitually use to cope and BA intervention might help strengthen the evidence for acceptability of BA, and any discrepancies might suggest possible adjustments that could further refine and strengthen the BA framework for young people.

### Search strategy, selection criteria, data extraction and analysis

Eligible RCT studies were systematically identified from the PracticeWise Evidence-Based Services (PWEBS) database. PWEBS is the largest repository of published youth (aged up to 21 years) mental health intervention research articles, identified and biannually updated through the systematic search of online databases and personal nominations submitted by trained consultants and professionals. Each article in the PWEBS database undergoes a rigorous double-coding and validation process to produce a standardized and structured interpretation of various intervention and outcome indicators [20, 21]. Since the standard PWEBS database focuses on study samples aged up to 21 years, to examine the evidence for the older age group (upto age of 24 years), we used a complementary transition age youth database. The database was under development at the time of our review, and studies were coded using the PWEBS system but not yet validated. The databases were accessed in June–August 2020.

A full breakdown of the detailed search strategy with SPIDER elements [22] used to identify relevant RCTs across two PWEBS databases is provided in Table 1. Based on Kanter’s systematic review [12], the standalone BA intervention was defined as an intervention that primarily used behavioral approaches, with activity scheduling and self-monitoring as essential intervention elements. Similarly, BA as an element in the multicomponent intervention was defined as  the combination of activity scheduling and self-monitoring elements. We excluded studies that examined universal interventions (i.e., interventions that are offered to all young people regardless of whether or not they are at risk for, or have, mental health problems), studies limited to institute specific samples, and studies where BA was an optional element in the intervention package.

**Table 1:** Search strategy with SPIDER elements used to identify quantitative and qualitative studies for systematic review

SPIDER Element	Search Strategy
Sample	Quantitative review: mean age between 14–24 years; study sample selected based on elevated symptoms or diagnosis of anxiety or depression. Qualitative review: mean age or majority of participants (50% or more) aged between 14–24 years; study sample selected based on elevated symptoms or diagnosis or self-reported lived experience of anxiety or depression
Phenomenon of Interest	Quantitative review: characteristics and outcomes of indicated prevention/treatment studies, where BA was included as the standalone intervention or else a constituent practice element of the multicomponent intervention protocol
	Qualitative review: Lived experience of the defined youth population of participating in the standalone BA intervention or multicomponent intervention where BA was highlighted as a theme; lived experience of coping among individuals from the defined youth population
Design	Quantitative review: randomized controlled trials
	Qualitative: interview or group-discussion studies using any analytic method
Evaluation	Quantitative review: validated measures of anxiety or depression
	Qualitative review: first-hand experiential accounts (including direct quotations) of intervention participants
Research type	Quantitative review: randomized comparisons of standalone intervention or multicomponent interventions against one or more control group(s)
	Qualitative review: qualitative studies, or mixed-methods studies which contained substantial qualitative data
Search terms	Quantitative review: Inclusion and exclusion of quantitative studies on the above criteria was determined based on information coded already in PracticeWise database
	Qualitative review- (i): Database-specific methods of using the following search terms were applied: (‘youth’ OR ‘young*’ OR ‘teen’ OR ‘adolescen*’ OR ‘minor’ OR ‘pube*’) AND activation (‘behavio* activation’ OR ‘activity schedul*’ OR ‘behavio* therapy’ OR ‘activity selection’) AND (‘focus group’ OR ‘interview’ OR ‘survey’ OR’report’) AND (‘feel’ OR ‘understand*’ OR ‘acceptab*’ OR ‘experience’) AND (‘qualitative’ OR ‘mixed method’ OR ‘thematic synthesis’)
	(ii): Database-specific methods of using the following search terms were applied: (‘youth’ OR ‘young*’ OR ‘teen’ OR ‘adolescen*’ OR ‘minor’ OR ‘pube*’) AND (‘anxiety’ OR ‘depression’ OR ‘transdiagnostic’ OR ‘common mental health’) AND (‘focus group’ OR ‘interview’ OR ‘survey’ OR’report’) AND (‘view’ OR ‘opinion’ OR ‘experience’ OR ‘cop*’ OR ‘attitude’ OR ‘strateg*’ OR ‘belie*) AND(‘qualitative’ OR ‘mixed method’ OR ‘thematic synthesis’)

Using the PWEBS coding system [21], indicators on sample, intervention, and outcomes were extracted for all eligible studies and summarized using narrative synthesis. The study outcome for the primary measure of depression and/or anxiety was categorized as ‘superior’, ‘valid equivalent’ or ‘non-superior’, based on the performance of the index intervention group in comparison to other study groups and the wider evidence for intervention in PWEBS database. The definition of these variables is provided in Table 2.

**Table 2:** Intervention and outcome indicators for the quantitative studies

Indicators	Definition
Intervention type	Comprised of indicated prevention and treatment interventions
	Indicated prevention were those that recruited participants based on an elevated score on a symptom measure
	Treatment intervention were those that recruited participants based on meeting the clinical criteria on diagnostic measures or clinical threshold on symptom measures
Format and modality	Whether the intervention was delivered in (1) group or individual format, (2) face-to-face or digitally, (3) involved parents or not
Dosage of session	The number of sessions intended to be held for the index intervention
Frequency of sessions	Frequency with which sessions were held: daily, weekly, biweekly, semiweekly
Duration of intervention	The minimum and maximum length of time from pre- to post- intervention
Provider	Highest education qualification for the providers in the index group (doctoral or non-doctoral)
Trainability	Extent to which others can be trained in an intervention; rated as high if the manual was available and treatment was delivered by non-doctoral-level practitioners; as moderate if the manual was available or treatment was delivered by non-doctoral-level practitioners; and as low if no manual was available and treatment delivered by doctoral-level practitioners only
Outcome status	Superior was applied when an intervention performed better than one or more other study groups (a psychosocial intervention, medication, combined psychosocial and medication, placebo, waitlist, no treatment, or other control groups) in a randomized trial on the primary outcome measure in the target symptom domain
	Valid equivalent was applied when an intervention had a qualifying tie with one or more evidence-based protocol in a randomized trial on the primary outcome measure in the target symptom domain
	Non-superior was applied when an intervention (i) performed worse than comparison groups, or (ii) performed equally against a non-evidence-based comparison group in a randomized trial on the primary outcome measure in the target symptom domain

PsycInfo, PubMed and Google Scholar research databases were systematically reviewed to identify qualitative and mixed-method studies that examined young people’s experiences with BA in standalone/ multicomponent intervention for depression and/or anxiety (SPIDER elements and search terms provided in the Table 1). We also reviewed studies that examined adaptive strategies habitually used by young people to effectively cope with depression and anxiety and the extent of their alignment with BA (search terms provided in the Table 1). We excluded purely quantitative studies, ethnography and observational studies and qualitative studies that gather information from caregivers or providers only. These databases were accessed through period of June–August 2020. The reference sections of the randomized trial papers identified through the PWEBS search were hand-searched to identify additional qualitative evaluations and authors of these papers were contacted to extract any qualitative data that might have been included as part of the randomized trials.

**Fig. 1:**
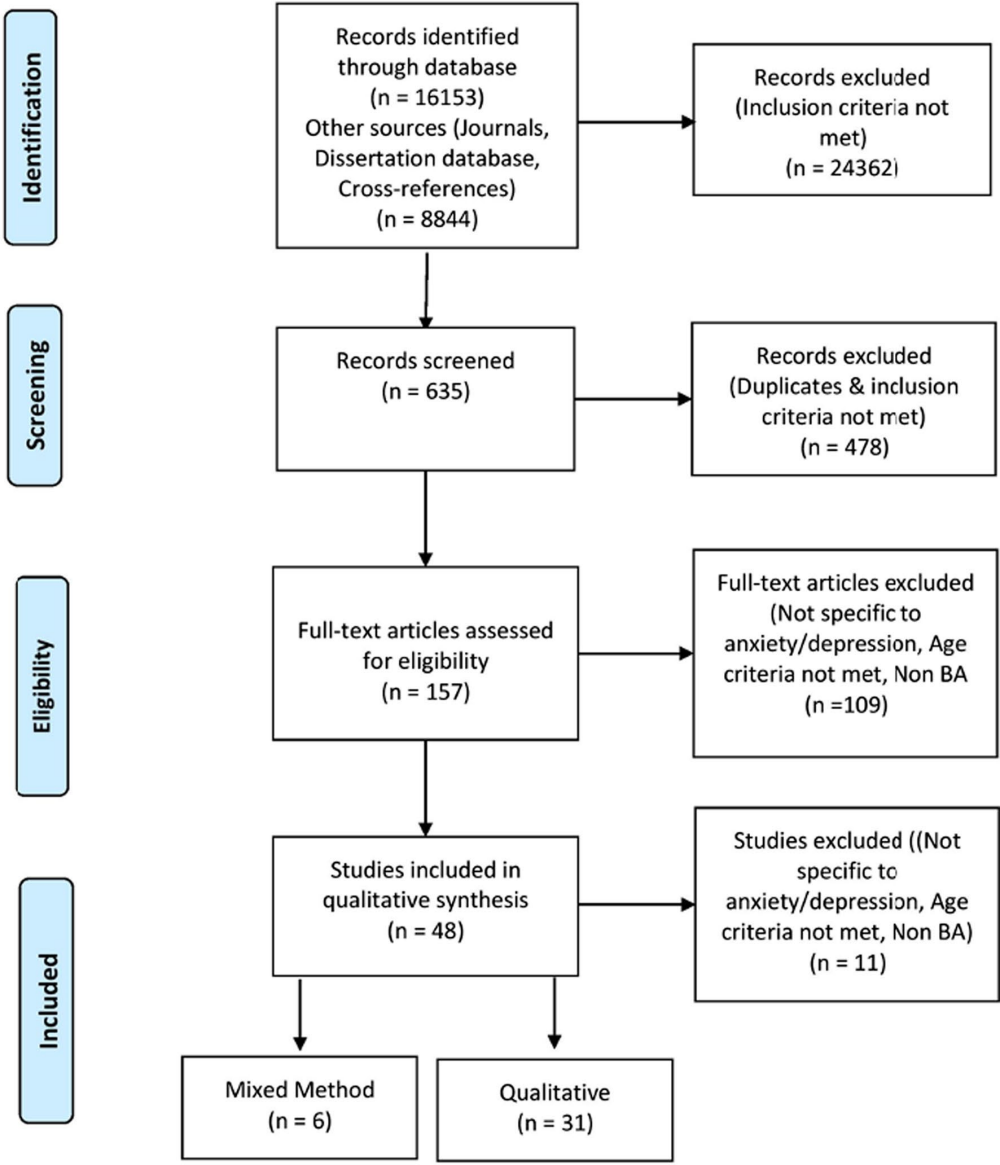
PRISMA flowchart for selection of studies of qualitative and mixed-method studies

The selection process, including search results and reasons for exclusion at each stage of screening are represented in the PRISMA flow diagram Fig. 1. The title and abstract of identified articles were screened for potential eligibility by two project researchers (RKV, TR). The shortlisted articles were read in full by the third researcher (MI) to determine if they met the eligibility criteria. For each eligible study, the sample characteristics, findings from results and discussion sections along with participant quotes, were extracted by one of the three researchers (MI, RKV, TR) and cross-checked by another project researcher before conducting analysis. Thematic synthesis was used for analyzing and summarizing data [23, 24]. This involved line-by-line coding of data from each article by two project researchers. Any discrepancies in coding at this stage were resolved through discussion. This was followed by identification of descriptive themes, and generation of analytical themes that extended upon the descriptive themes. NVivo 12 [25] was used for coding data and grouping it into themes.

### Risk of bias and study quality

Eligible RCT studies were assessed for quality using the Cochrane Collaboration’s Risk of Bias (RoB) assessment tool [26]. The following domains were rated for bias: (1) random sequence generation (selection bias), (2) allocation concealment (selection bias), (3) blinding of outcome assessment (detection bias), and (4) incomplete outcome data (attrition bias). As per the tool guidelines, the bias for each domain was rated as high, low, or unclear. Studies where self-report tools were used, detection bias was marked as not applicable (NA) and studies where intention-to-treat analysis was used, attrition bias was rated as low. In line with previous reviews [27, 28], biases due to selective reporting (trial registry details were not available for most studies) and blinding of participants/ personnel (studies of psychological interventions are typically not able to blind participants and personnel) were not included.

Mixed Methods Appraisal Tool (MMAT) [29] was used for quality assessment of the qualitative and mixed-method studies. Each study was rated on seven domains: clarity of stated research questions, appropriateness of the chosen approach to address the research question, adequacy of data collection methods, derivation of findings from data, quality of analyzed results, coherence between data sources and results, and interpretation of finding. For each domain, a three-point rating scale was used : 0 (low degree of confidence), 0.5 (mixed/unclear), or 1 (high degree of confidence). The maximum possible total score for each study was 7, with a higher score indicating good confidence in study findings.

For bias and quality assessments, each study was independently rated by two project researchers. Disagreements were resolved by discussion until consensus was reached, and if required, a third independent researcher went through the full text of the disputed study to check each domain for inconsistencies.

### Consultations with youth advisors

Engaging individuals who are the focus of research into the research process can lead to better outcomes and ultimately better service, in particular for young people, whose “voices” are often absent from the published literature [30, 31]. In line with the principles of participatory research, consultative workshops were held with a Youth Advisor Group (YAG) to draw inferences from published evidence and identify research priorities, specifically for LMICs like India. The youth advisors were recruited through the “It’sOkToTalk” initiative at Sangath, India (http://itsoktotalk.in/get-involved/). A digital flyer highlighting the workshop’s objectives and role of youth advisors was circulated through the website, inviting young people across India with self-identified lived experience of depression and anxiety to contact the project researchers (MI and TR) through an email. Interested young people were asked to complete a demographic form and assent/ consent form to participate as advisors. For young people below the age of 18 years, additional written consent was obtained from their parent/guardian. The demographic details of the ten members of the youth advisor group are given in the supplement section (Additional file 1). A total of three, 2-h long online workshops (using a secure video-conferencing platform) were organized with youth advisors. The consultations with YAG were structured around brief presentations on BA theory and intervention, preliminary findings from the review, and design of the dissemination materials. These presentations were followed by semi-structured group discussions, facilitated by project researchers (KM and MI), to examine advisors’ perception about the utility of BA in young people, the credibility of the preliminary evidence synthesis, potential refinements, and implications for further research. The responses obtained from the youth advisors were analyzed thematically using the same framework that was developed to synthesize the qualitative studies.

## Results

### Narrative synthesis of data from the review of quantitative studies

A total of 23 RCT eligible studies were identified, three (13%) studies examined standalone BA interventions, and 20 studies (87%) examined multicomponent interventions with BA. The details of these studies on selected variables are presented in Table 3.

**Table 3:** Study details for quantitative studies on BA for depression and anxiety (listed in alphabetical order)

First Author	Year	Sample	Recruitment settings; Site	Index group & its composition	Intervention type	Primary target	Index Sample size	Mean Age	Female %	Comparator	Outcome
**BA as standalone intervention (n = 3)**											
Goodyer^*^^	2017	470	Clinic; UK	Brief psychosocial intervention comprising 12 elements	Treatment	Depression	155	15.60	74	Active (CBT, STPP)	Non-superior to both comparators
McCauley*^#	2016	60	Clinic; USA	BA comprising 8 elements	Treatment	Depression	27	15.17	63	Active (CBT/IPT)	Non-superior
Takagaki^^#^	2016	118	University; Japan	BA comprising 6 elements	Indicated	Depression	62	18.23	39	Clinical monitoring	Superior
**BA in multicomponent intervention (n = 22)**											
Brent^	1997	107	Clinic, Community; USA	CBT comprising 14 elements	Treatment	Depression	37	15.70	76	Active (Family therapy, non-directive counselling)	Superior than both active comparators
Burton	2007	145	School, University; USA	CBT comprising 11 elements	Indicated	Depression	74	18.60	100	Waitlist	Superior
Clarke^^^	1999	123	Community; USA	CBT with adolescents comprising 17 elements	Indicated	Depression	37	16.20	71	Waitlist & Active (CBT with adolescents and parents)	Superior than waitlist comparator; valid equivalence with active comparator
				CBT with adolescents and parents comprising 18 elements	Indicated	Depression	32	16.20	71	Waitlist & Active (CBT with adolescents only)	Superior than waitlist comparator; valid equivalence with active comparator
Deady	2016	104	Community; Australia	Internet-based CBT comprising 10 elements	Indicated	Depression	60	21.85	60	Attention control	Superior
Ip^*^	2016	257	School; China	Internet-based CBT comprising 8 elements	Treatment	Depression	123	14.64	70	Attention control	Non-superior
Kobak^^^	2015	76	Community; NA	Technology enhanced CBT comprising 8 elements	Treatment	Depression	35	15.40	66	Active	Non-superior
Lewinsohn^*^	1990	59	Community; USA	CBT with adolescents comprising 17 elements	Treatment	Depression	19	16.15	62	Waitlist & Active (CBT with adolescents and parents)	Superior than waitlist comparator; non-superior with active comparator
				CBT with adolescents and parents comprising 18 elements	Treatment	Depression	21	16.26	53	Waitlist & Active (CBT with adolescents and parents)	Superior than waitlist comparator; non-superior with active comparator
Ranney	2018	116	Clinic; USA	Text-based CBT comprising 7 elements	Treatment	Depression	58	14.83	59	Attention control	Non-superior
Reynolds	1986	30	School; NA	CBT comprising 9 elements	Indicated	Depression	6	15.65	63	Waitlist & Active (relaxation)	Superior than waitlist comparator; non-superior with active comparator
Rohde^	2004	93	Community; USA	CBT comprising 17 elements	Treatment	Depression	44	15.10	60	Attention control	Superior
Rosselló	1999	71	School; USA	CBT comprising 9 elements	Indicated	Depression	21	15.00	54	Waitlist & Active (IPT)	Superior than waitlist comparator; valid equivalence with active comparator
Rosselló ^^^	2008	112	School; USA	CBT comprising 9 elements	Treatment	Depression	52	14.52	55	Active (IPT)	Superior
Shirk	2014	43	Clinic; NA	CBT + Mindfulness comprising 8 elements	Treatment	Depression	15	15.25	85	Active (TAU)	Non-superior
Stasiak^	2012	34	School; New Zealand	Computerized CBT comprising 9 elements	Treatment	Depression	16	15.47	53	Attention control	Superior
Stice	2007	225	School; USA	CBT comprising	Indicated	Depression	50	18.40	70	Waitlist & Active (Supportive therapy, Journaling, Bibliotherapy, Expressive writing)	Superior than waitlist comparator; valid equivalence with bibliotherapy; non-superior compared to other active comparators
TADS Team^^^	2004	439	Community; USA	CBT comprising 10 elements	Treatment	Depression	111	14.60	54	Active (medication with CBT, medication alone) and Attention control	Non-superior to all comparators
Tandon	2011	61	Community; USA	Mother and Baby CBT comprising 7 elements	Treatment	Depression	32	24.10	100	Active (TAU)	Superior
Topooco^*^	2018	71	Community; NA	Internet-Based CBT comprising 8 elements	Treatment	Depression	33	17.20	94	Attention control	Superior
van der Zanden^*^	2012	244	Community; Netherlands	Internet-Based CBT comprising 6 elements	Indicated	Depression	96	20.80	84	Waitlist	Superior
Wright^*^	2017	91	Clinic; UK	Computerized CBT comprising 8 elements	Treatment	Depression	25	15.50	73	Attention control	Non-superior

^*^Included anxiety as a secondary outcome, ^^^Included functioning as a secondary outcome, ^#^Included activation as a secondary outcome, $ In all trials with non-superior outcomes, there were no statistically significant differences between the index and the non-evidence-based comparative group. None of the trials with non-superior outcomes used the inferiority design.

UK- United Kingdom, USA- United States of America, BA-Behavior Activation, CBT-Cognitive Behavior Therapy, IPT- Interpersonal Psychotherapy, TAU- Treatment as usual, NA- Not Available

The RoB in most RCT studies was moderate, with biases related to allocation by an independent (third) party (13 out of 23 studies, 57%) and masked assessment procedure being most frequent (18 out of 23 studies, 78%). The RoB ratings for individual studies are given in Box 1.

**Figure:**
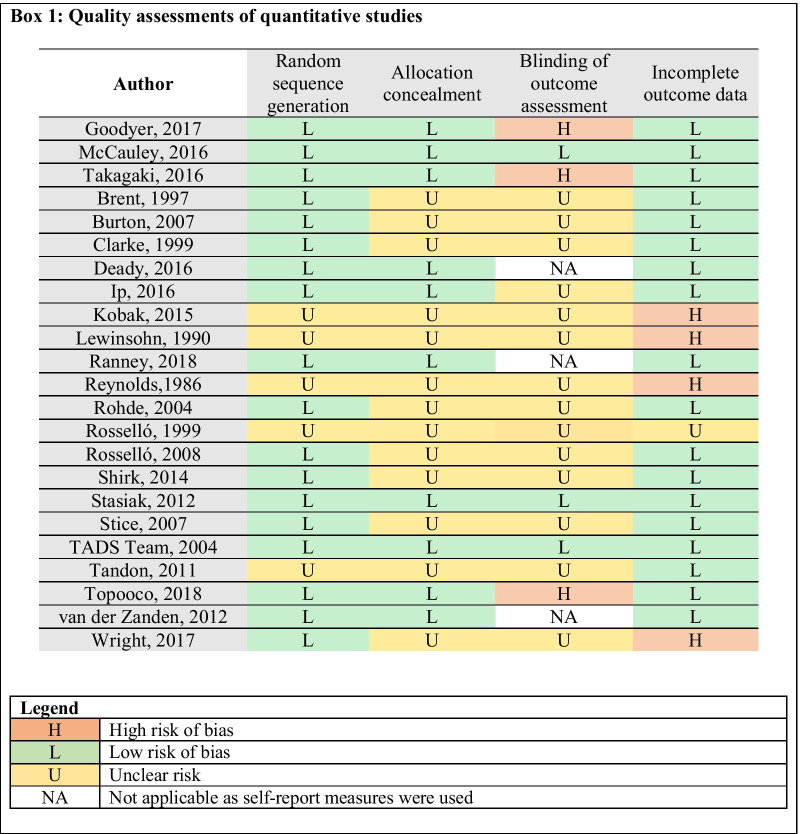

*Study characteristics, intervention characteristics and effectiveness of standalone BA interventions.* The sample size of the eligible studies varied between 60 to 470, with a total of 648 participants across three studies, recruited from clinical and university settings. All three RCTs were conducted in high-income countries (HICs). There was a greater representation of adolescents (compared with young adults) and female participants across studies (Table 3).

The number of intervention elements in standalone BA intervention protocol varied across three studies (Range = 6–12, Table 3). BA was delivered as a brief intervention (5 sessions over 5 weeks) in one study that used psychoeducation, activity scheduling, goal setting, self-monitoring, motivation enhancement and relapse prevention to increase and maintain exposure to positive reinforcements for healthy behavior. This intervention did not focus on avoidant behaviors [32]. In the other two studies, BA was delivered as a relatively longer treatment (12–14 sessions) [33, 34] that incorporated a number of additional skills (e.g., problem-solving, functional analysis for countering avoidance, see Box 2) to address avoidance processes and other barriers that interfered with positive behavioral changes. All three interventions were delivered face-to-face, in an individual format, by specialist providers; one intervention was delivered simultaneously in individual and group format. The intervention delivery was supported by a workbook and structured material in two of three interventions [32, 34]. In all three interventions, collaboratively developed and shared home practice assignments were used to facilitate BA skills. The intervention protocol completion rate was high across all three studies (98.4% [32], 83% [33] and 83% [34]), with no attrition bias. The other details on format, providers, trainability, session dosage are summarized in Box 2.

**Figure:**
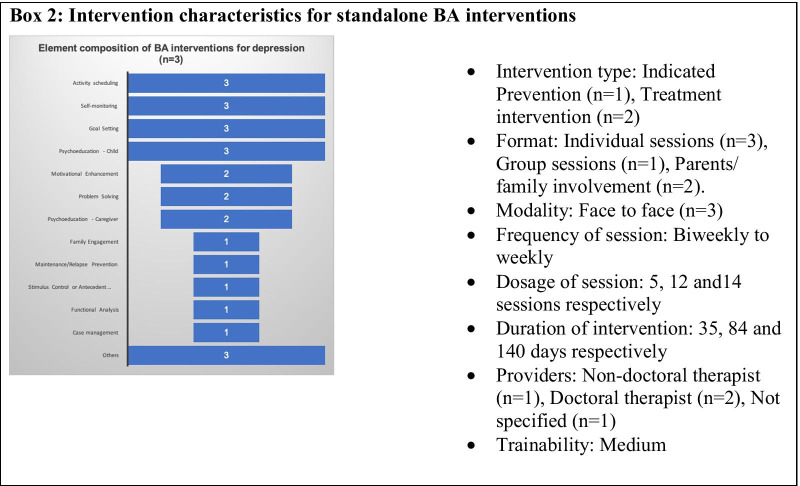

Depression was the primary target problem in all three studies. Anxiety was a secondary outcome in two studies [33, 34]. Additionally, functioning and activation were assessed as secondary outcomes in three [32–34] and two studies [32, 34], respectively. The details of outcome measures are given in the Additional file 2. BA interventions were compared with four groups across three studies and achieved superior outcomes in one comparison (25%) and non-superior outcomes in the three comparisons (75%) (Table 4). In the study that showed superior outcome, the brief, indicated BA intervention was compared to an inactive control group (i.e., clinical monitoring) [34]. The study also found significant intervention effects on measures of activation and functioning. In the remaining two studies [33, 34], where the relatively longer BA treatments were compared to active interventions, including cognitive behavioral therapy, short-term psychoanalytical therapy and evidence-based practice, the outcomes did not differ significantly between the BA and comparison groups (‘non-superior’) for depression [33, 34], functioning [33, 34] and activation [34]. Across three studies, there was a lack of sufficient data to establish valid outcomes on anxiety measures.

**Table 4:** Outcome of RCT index group in comparison to the other study groups

Type of comparator group	No. of comparator groups	Study Sample	Superior/ equivalence – non-superior (n)	Characteristic of intervention with superior/ equivalence outcome (n)	Characteristic of intervention with non-superior outcome (n)
**BA as standalone intervention (n = 3)**					
Waitlist/ no treatment/ attention control	–	–	–	–	–
Clinical monitoring	1	118	1–0	Indicated (1); Treatment (0)	Indicated (NA); Treatment (NA)
				Individual (1); Group (1)	Individual (NA); Group (NA)
				Duration: 35 days	Duration: NA
Active treatment	3	530	0–3	Indicated (NA); Treatment (NA)	Indicated (0); Treatment (3)
				Individual (NA); Group (NA)	Individual (3); Group (0)
					Parent involved (3)
				Duration: NA	Duration: 84–140 days
Any comparator	4	648	1-3	Indicated (1); Treatment (0)	Indicated (0); Treatment (3)
				Individual (1); Group (1)	Individual (2); Group (0)
					Parent involved (3)
				Duration: 35 days	Duration: 84–140 days
**BA in multicomponent intervention (n = 22)**					
No treatment/ clinical monitoring	–	–	–	–	–
Waitlist	9	897	9-0	Indicated (7); Treatment (2)	Indicated (NA); Treatment (NA)
				Individual (1); Group (8)	Individual (NA); Group (NA)
				Parent involved (2); Digital (1)	Duration: NA
				Median Duration = 49 days (Range: 28–84)	
Attention control*	8	1205	4-4	Indicated (1); Treatment (3)	Indicated (0); Treatment (4)
				Individual (3); Group (1)	Individual (4); Group (0)
				Digital (3)	Digital (3)
				Median Duration = 56 days (Range: 28–70)	Median Duration = 56 days (Range: 28–84)
Active treatment*	18	1346	8-10	Indicated (5); Treatment (3)	Indicated (3); Treatment (7)
				Individual (3); Group (6)	Individual (5); Group (5)
				Parent involved (2)	Parent involved (1)
				Median Duration = 56 days (Range: 28–112)	Median Duration = 49 days (Range: 28–84)
Any comparator	35	2501	21–14	Indicated (13); Treatment (8)	Indicated (3); Treatment (11)
				Individual (7); Group (15)	Individual (9); Group (5)
				Parent involved (4); Digital (4)	Parent involved (1); Digital (3)
				Median Duration = 49 days (Range: 28–84)	Median Duration = 56 days (Range: 28–84)

^*^ For one trial, the intervention duration data was not available and another one explicitly stated that sessions were intentionally varied and, therefore, these two trials were not included in the calculation of median duration

NA Not applicable

*Study characteristics, intervention characteristics and effectiveness of multicomponent interventions with BA elements.* The sample size of the eligible studies varied from 30 to 439 with a total of 2501 participants across 20 studies, recruited from varied settings (school, community, clinics), mostly in high-income countries (HICs). Similar to previous set of studies, there was a greater representation of adolescents and female participants across studies*.*

The multicomponent interventions were fairly broad and complex, comprising on average 11 (Range 7 to 18; SD = 4.08) intervention elements. The elements of multicomponent interventions are shown in Box 3. As techniques of BA, these interventions typically included generating a list of pleasurable activities, engaging in activities as per the jointly agreed schedule and monitoring its impact on their mood. Additionally, these multicomponent interventions included skill training elements (e.g., problem-solving [n = 12], social skills [n = 10], communication [n = 6] and assertiveness [n = 5]) designed to promote and overcome barriers to changes in targeted cognitions, behaviors and/or feelings. With exception of cognitive elements, most of the other elements were similar to those present in long formats of standalone BA interventions, as described previously [1]. The intervention protocol completion rate varied between 62 to 100%, with a higher attrition rate in digitally delivered intervention. The other details on format, providers, trainability, session dosage are summarized in Box 3.

**Figure:**
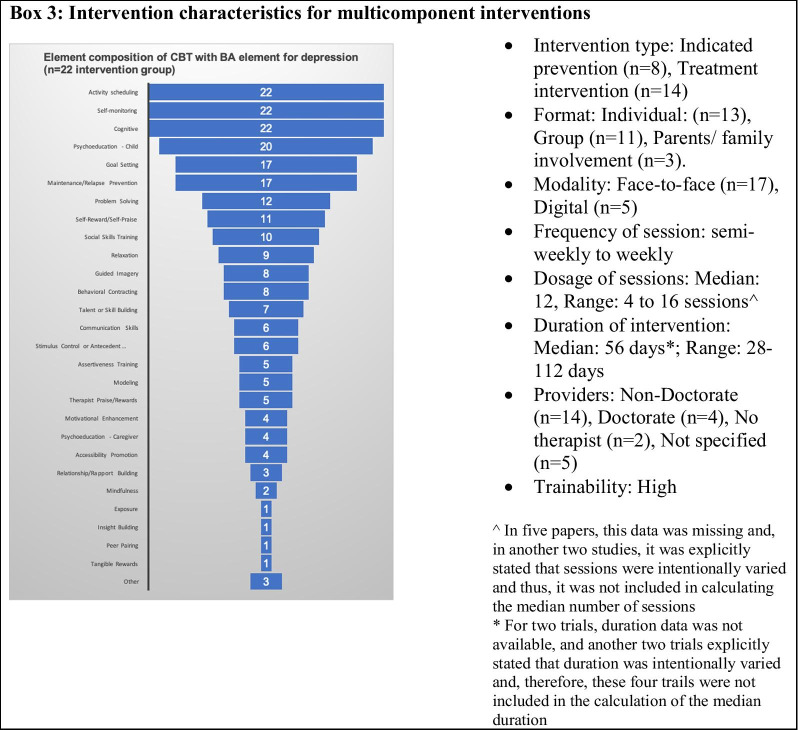

Depression was the primary target problem in all 20 studies, and anxiety was a secondary outcome in five studies [35–39]. Additionally, functioning was assessed in seven studies [40–46]. The details of outcome measures used for assessing depression, anxiety, and functioning are given in the Additional file 2. The multicomponent interventions were compared with 35 groups and recorded a superior or equivalent outcome in 21 (60%) comparisons on a measure of depression (Table 4). Outcomes for depression were more favorable when the multicomponent interventions were compared with the waitlist; comparisons of the multicomponent interventions with the active and attention control groups mostly resulted in a lack of significant difference in outcomes. Multicomponent interventions targeting subthreshold depression (indicated interventions) and delivered in group format were more likely to record a superior or equivalent outcome. There was no difference in the intervention length between multicomponent interventions with superior or valid equivalent outcomes and interventions with non-superior outcomes. Superior or valid equivalent outcomes for depression were accompanied by significant improvement in secondary measures of functioning [41, 43] and anxiety in a few studies [37]. None of these multicomponent studies included any focal measure of activation.

### Thematic synthesis of data from qualitative studies and YAG

A total of 37 eligible studies were identified through two search strategies (Table 1). Three studies [47–49] examined young people’s experience of participating in an intervention with BA: one study [47] focused on 14-session standalone BA intervention that was later evaluated by McCauley and colleagues in a trial [34]; the second study [48] examined a 10-session multicomponent BA intervention, similar to the intervention protocol evaluated by Clarke and colleagues [42]; and the third study [49] examined experiences with internet-based multicomponent BA intervention that was later evaluated by Ip and colleagues in a RCT [36]. The remaining 34 studies [50–83] examined adaptive coping in young people with lived experience of depression and anxiety. The sample size in these studies varied from 8 to 251, with a total of 1524 youth, all from HICs, with an average age of 17.16 years (SD = 1.86). The sample included 65% females. Of 37 studies, 29 were specific to depression, three focused on anxiety, and five focused on both. The other details of these studies on selected variables are presented in Table 5.

**Table 5:** Study details for qualitative studies (listed in alphabetical order)

First author	Year	Sample size	Recruitment settings & site	Conditions included	Age range (Mean)	Female %	Data & analysis method	Key themes
**Studies on BA (n = 3)**								
Arnott ^+^	2020	8	School, UK	Depression	12–15 (14.06)	50	Interviews, TA	Structure and content acceptable; goal-oriented activities liked the best; homework assignments were helpful; positive impact on mood and functioning; non-specialist therapist guidance appreciated; staggering sessions/ top-up BA session at the end
Bru	2013	10	Clinic, Norway	Depression	17–20 (18.40)	80	Interviews, TA	Scheduling pleasurable activities increased awareness of what makes one happy; psychoeducation was easy to understand, but a few found the emphasis on individual responsibility unacceptable; homework assignments were easy to understand, but some thought it was time-consuming and effortful; suggestion to use simpler language in session and assignments was emphasized
Iloabachie ^+^	2011	83	Clinical and community, USA	Depression	14–21 (17.40)	56	Survey and Interviews, TA based on GT	Participants liked BA; positive gains were identified such as better understanding of mental health, sense of person control, better communication with family, decreased acting out behaviors and scheduling of healthy behaviors
**Studies on coping (n = 34)**								
Al-Khattab	2016	22	Community, USA	Depression	18–21 (20.10)	45	Interviews, CA	Behavioral (e.g., engaging in spiritual activities), and social strategies (e.g., support from others) were helpful
Aselton	2012	13	University, UK	Depression	19–22 (NA)	62	Online interviews, TA	Behavioral (e.g., engaging in hobbies, sports, journaling), and social strategies were helpful
Bluhm	2014	37	University, Australia	Depression & Anxiety	18–24 (20.60)	73	Interviews, TA	Social strategies and self-management were helpful
Boyd ^+^	2011	201	School, Australia	Depression	11–18 (NA)	63	Open-ended survey, CA	Self- acceptance was helpful
Breland-Noble	2010	28	Community, USA	Depression	11–17 (NA)	NA	FGD and Interviews, GT	Social strategies, self-management were helpful
Breland-Noble	2015	28	Community, USA	Depression	11–17 (NA)	NA	FGD and Interviews, TA	Behavioral strategies (e.g., prayers) and social strategies (e.g., social support from religious leaders) were helpful
Chernomas ^+^	2013	251	University, Canada	Depression & Anxiety	21–35 (22.40*)	NA	Open ended survey, TA	Behavioral strategies (e.g., running, yoga, time management, spiritual activities), social strategies (e.g., social interaction), and cognitive strategies were helpful
Dundon^**^	2006	107	NA	Depression	13–20 (NA)	84*	FGD and Interviews, Multi-method	Behavioral strategies, cognitive strategies, social strategies and self-management strategies (e.g., acceptance, taking self-initiative to improve functioning) were helpful
Farmer	2002	5	University, USA	Depression	13–17 (NA)	60	Interview, PA	Behavioral strategies (e.g., engaging in religious activities), social strategies, and self-care were helpful
Fornos	2005	65	School, USA	Depression	13–18 (15.60)	NA	FGD, TA	Social strategies were helpful
Grob	2002	38	Community, USA	Depression	18–29 (NA)	50	Interviews, TA based on GT	Behavioral strategies, cognitive strategies (e.g., positive thinking, challenging negative thoughts), social strategies (e.g., connecting those with mental health issues), self-acceptance (e.g., growth-promoting attitude) were helpful
Hannor-Walker	2008	10	Clinic, USA	Depression	14–17 (15.60*)	60	Interviews, CA	Social strategies (e.g., support from others) were helpful
Kuwabara	2007	15	Community, USA	Depression	18–25 (23.0)	67	Interviews, TA based on GT	Social strategies (e.g., support from others) were helpful
Martínez-Hernáez^+^	2016	105	Community, Spain	Depression	17–21 (NA)	69	Open ended survey and FGD, TA	Social strategies (support from others) were helpful
Martínez-Hernáez	2014	105	Community, Spain	Depression & Anxiety	17–21 (NA)	69	FGD and Interviews, TA based on GT	Behavioural strategies (scheduling time and activities) and social strategies were helpful
McCarthy	2008	9	University, USA	Depression	20–23 (20.20)	78	Interviews, TA	Social strategies (support from others) were helpful
McCann	2012	26	Clinic, Australia	Depression	16–22 (18.0)	NA	Interviews, IPA	Social strategies (support from others) were helpful
Morey-Nase	2019	11	Clinic, Australia	Depression	15–25 (21.4)	64	Interviews, TA	Behavioral strategies, self-management and acceptance and social strategies were helpful
Moses^+^	2009	54	Clinic, USA	Depression & Anxiety	12–18 (14.60)	37	Interviews, CA	Self-management and acceptance were helpful
Ofonedu	2013	10	Clinic, USA	Depression	13–17 (NA)	60	Interviews, PA	Self-management strategies and social strategies were helpful
Oliver	2015	7	Clinic, UK	Depression	–16–18 (16.85)	71	Interviews, IPA	Self-management strategies, social strategies (seeking support, social activities), behavioral strategies (pleasurable activities, important activities) were helpful
Özkul & Günüşen	2020	21	School, Turkey	Depression	14–15 (14.33)	67	Interviews, CA	Behavioral strategies (e.g., engagement in hobbies, scheduling activities), social strategies, self-management strategies were helpful
Recto & Champion	2018	20	School, USA	Depression	15–19 (17.15)	100	Interviews, CA	Self- management strategies was helpful
Ross	2003	48	Community, USA	Depression	13–22 (NA)	NA	FGD, TA	Social strategies were helpful
Ross	2015	6	Clinic, Canada	Anxiety	18–22 (NA)	100	Interviews, TA and case-study	Behavioral strategies (e.g., journaling, goal setting, engaging in enjoyable activities), social strategies, cognitive strategies (e.g., positive self-talk), self-management strategies were helpful
Sabiston	2007	31	Community, Canada	Anxiety	13–18 (15.58)	100	Interviews, TA	Behavioral strategies (e.g., planning healthy eating), cognitive strategies (e.g., distraction, reappraisal), self-management strategies were helpful
Sam	2019	9	University, USA	Depression	Above 18	NA	Interviews, Frame analysis based on GT	Social strategies were helpful
Simonds	2014	9	Clinic, UK	Depression & Anxiety	14–16 (NA)	78	Interviews, TA	Behavioral strategies (e.g., goal setting) and self-management strategies (e.g., awareness of and agency over self) were helpful
Weitkamp	2016	6	Clinic, Germany	Depression	15–19 (NA)	83	Interviews, IPA	Social strategies (e.g., indulging in social activities) were helpful
Wisdom	2004	15	School, USA	Depression	14–19 (16.30)	53.3	FGD, TA	Self-management strategies (e.g., positive labeling for their condition)
Wisdom	2007	15	Clinic, USA	Depression	14–19 (16.30)	53.3	Interviews, TA	Social strategies (e.g., connected to those with similar experiences) were helpful
Wisdom	2006	14	Clinic, USA	Depression	14–19 (16.3)	50	FGD and Interviews, CA	Behavioral strategies (e.g., seeking pleasurable activities), cognitive strategies, social strategies, self-management and acceptance were helpful
Woodgate	2006	14	Clinic, Canada	Depression	13.5–18 (16.0)	78.5	FGD and Interviews, HP	Cognitive strategies (e.g., positive thinking), social strategies (e.g., connecting with a network of those with mental health issues), self-management and acceptance were helpful
Woodgate	2020	58	Clinic, Canada	Anxiety	10–22 (14.50)	NA	Ecomap and Interviews, HP	Self-management strategies were helpful

^*^Inferred from the data—Not explicitly mentioned, **Metasynthesis, ^+^Mixed-method studies

UK—United Kingdom, USA—United States of America, FGD—Focus Group Discussion, TA—Thematic Analysis, CA—Content Analysis, GT—Grounded Theory, IPA—Interpretative Phenomenological Analysis, PA—Phenomenological Analysis, HP—Hermeneutic Phenomenology, NA—Not Available

For qualitative and mixed-method studies, the quality of most of the included studies was good (31 of 37 studies [84%] scored in full on the MMAT domains). Six studies had lower scores due to biases such as poor coherence between data sources, lack of clarity on the findings being adequately derived from data, and lack of adequate integration of study components to answer the research questions. The MMAT ratings for individual studies are given in Box 4.

**Figure:**
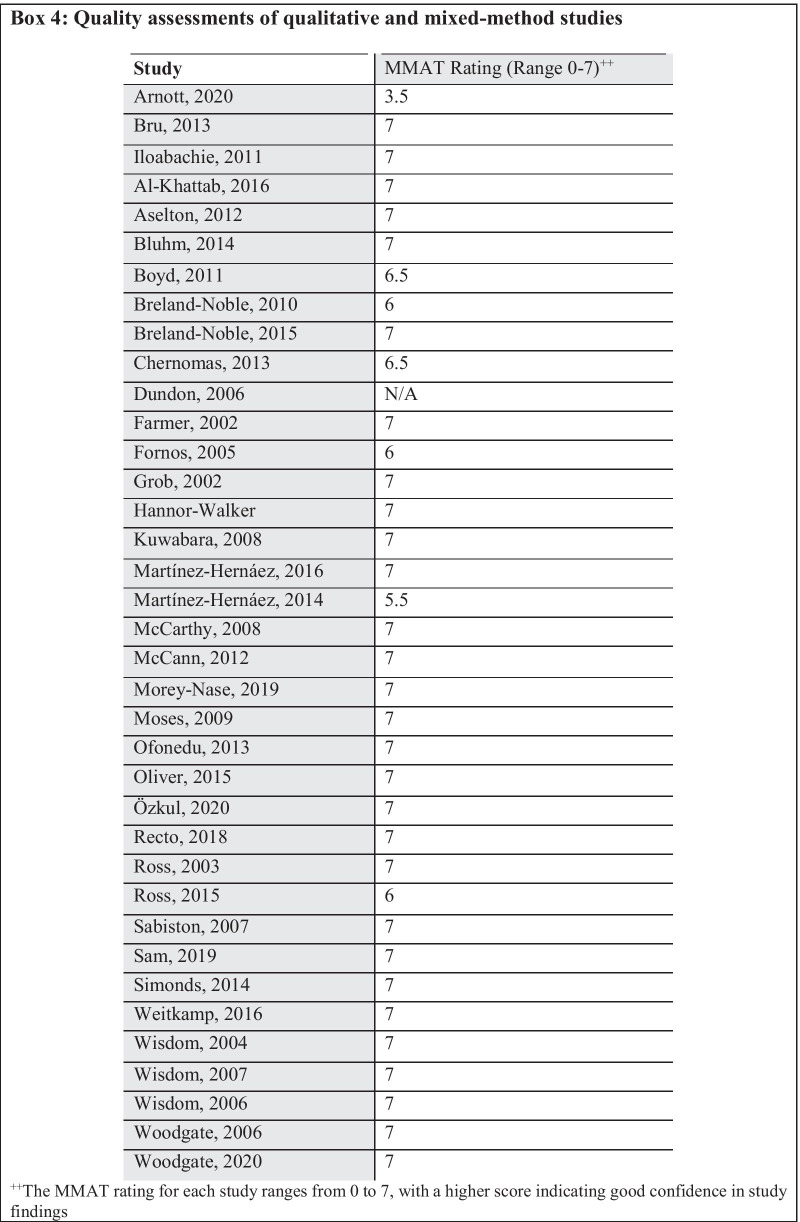

The descriptive themes from the thematic synthesis of qualitative studies are summarized and collated under the following analytic themes: (1) Positive aspects (2) Challenging aspects (3) Aspects for improvement (4) BA's alignment with habitual coping (5) Additional strategies to optimize BA (Table 6). The themes identified from workshops with YAG members were also collated using the same framework and are presented in Table 7.

**Table 6:** Results of thematic synthesis from qualitative studies

Analytic themes	Descriptive themes	N*	Sample quotes [study’s citation]
**Themes from lived experience literature on BA intervention**			
Positive Aspects	Appropriate format	13	“I would definitely have chosen it [to have the homework assignments], because you get a better understanding of what the course is about [by having homework]. And you can repeat what you have learned at home, so that you learn it better.” [48]
			“Talked about what I wanted to talk about (during sessions).” [47]
	Enhanced mood	5	“I can understand how my behavior and my habits can affect my mood, I can change my depression by changing my behavior.” [49]
	Improved functioning	9	“I spend much more time together with other people. Before the course, I just went home and straight to bed. Now, I force myself to socialize with friends and acquaintances … it makes me happy, and I want to do more of the things that make me feel that way …” [48]
Challenging aspects	Difficulty sustaining change	6	“When you’re home, you want to do other things. You don’t want to do assignments, which can be boring. When I’m home, I’d rather read a book of my own choosing.” [48]
			"They [the homework assignments] were okay, there was nothing negative about them. It just took a lot of effort doing them.”[48]
	Personal responsibility	3	“I felt I got a lot of responsibility for why I was depressed. And in a way, you were sitting there and saying, ‘I can’t help it, I didn’t do it on purpose’.” [48]
Aspects for improvement	Staggered sessions	5	“Top- up BA sessions following the end of treatment; this may help bridge the gap between reliance on the therapist and independence at home.” [47]
	Simplified language	2	“Maybe use simpler language, so one can understand it better. I have noticed that there are many technical terms in the assignments.” [48]
**Themes from lived experience literature on adaptive habitual coping**			
BA's alignment with habitual coping	Behavioural strategies	150	“I set myself reminders in my phone (*for my academic activities)*, I used the calendar on my phone, I might physically write things down” [54]
			“I found it very helpful to separate myself from whatever makes me feel depressed and relax and listen to music. Listening to music has really helped calm me down and forget about my worries.” [51]
			“I think that…religion might help somebody go to a counsellor or therapist because um…what if God might be telling them that how maybe if they were thinking of hurting they self or killing their self, so they need a counsellor.” [52]
	Cognitive restructuring	20	“So, I tell myself ‘you’re okay, you’re okay. Stay right here. What can you deal with right now? What is it that is stressing you out? Then you answer back. You have this but you have time. You’ve always done it before.’ I just reassure myself” [55]
Additional strategies to optimize BA	Role of social support	173	“My friend reminded me that I wasn’t alone. On occasions, she didn’t necessarily say that much, but when I was having an anxiety attack, she would give me a hug and we would just sit, and she would help me through it and help me breathe.” [55]
			“My mother would always come to me whenever a problem was going on. I don’t know how. I wouldn’t even tell her anything was going on. She would just know something was not right.” [50]
	Self- acceptance and self-care	85	“When I was younger I just felt stupid, I felt like my feelings weren’t valid... and now I’m in a place where I could really identify... like I do struggle with things and that’s okay. But there’s a way I could struggle that’s helpful not hurtful to myself and to my relationships.” [53]
			“I don’t get embarrassed like if I did something wrong. I just get up and try again. Whereas before I wouldn’t.” [56]

*N refers to frequency with which the theme was mentioned across studies

**Table 7:** Themes from workshops with the Youth Advisory Group (YAG)

Theme	Sub-themes	N*	Quotes
Positive aspects	Appropriate format	8	“Can be customized to an individual’s needs”
			“Gives a routine, even during Covid-19 which gives a sense of comfort and normalcy”
			“Get satisfaction after completing a set number of things/tasks, talk therapy is helpful but doesn’t provide these concrete aspects and makes me unsure of my progress”
			“Therapist guidance and reminders would be helpful”
	Improvement in symptoms, coping and functioning	4	“Doing one thing does not do away depression, but helps cope; e.g., just taking a shower makes the entire day better.”
			“Better coping, fewer anxiety attacks, being more social”
Challenging aspects	Difficulty sustaining change	9	“Temporary- If I do something and feel better now, but how would I feel better over time and contribute to overall wellbeing?”
			“Need to find foundational motivation within self for BA activities. Not everyone can do that, might be too much work, which may not work for everyone, depends on a person’s initial capacity and functioning. Every therapy doesn't work for everyone.”
	Unintended adverse emotions	3	“Personal responsibility, not being able to complete activities might induce guilt/anxiety”
	Lacks sensitivity to social and contextual factors	4	“Focus is on individual as responsible – needs cognizance of structures & institutions that cause psychological distress at a systemic level”
			“Also depends on the environment- access to some activities, stigma based on gender identity (e.g.: a transman attending the gym, which restroom to use safely etc.)”
Additional strategies	Structure of intervention	11	“Give more clarity on key elements in BA, does activity scheduling mean you have to keep adding on more activities until it works if the first few activities don’t work?”
			“Activities that are more personal rather than pre-decided would be helpful.”
			“Consider role of social support, spiritual coping, mindfulness etc.”
			“Buddy system or accountability partners included in therapy plan”
	Education and dissemination of BA	8	“Also provide MH [mental health] resources for young people, their friends and family is needed”
			“If not worded properly, BA can seem like we are blaming the person for having a mental illness but what it really means is that: while the fact that we are depressed/have anxiety is not our fault, how we deal with it is up to us. Depression and anxiety, ultimately, are not crippling or out of our control. Again, not trying to paint this as a mind over matter sort of thing but rather a ‘this is something we can learn to cope with' thing.”

N refers to the number of Youth Advisory Group (YAG) members who mentioned the particular theme

Evidence from the coping literature indicated that young people habitually used a number of behavioral strategies (over cognitive ones) in their natural environment to alleviate depression and, to some extent, anxiety. These strategies, in principle, were similar to BA intervention strategies. These strategies included engaging in pleasurable activities, preparing schedules and managing study time, setting reminders for daily tasks, engaging in religious and spiritual activities. Themes from studies on BA interventions indicated that participants liked the structure and content of BA programs and recognized its positive impact on mood and functioning (Table 6). YAG members also acknowledged that the strength of BA is in it being a proactive technique that could be “customized to an individual’s needs”. YAG members highlighted that unlike “talk therapy”, the impetus on activities could help young people feel more in control, improve self-confidence, and provide satisfaction with treatment. Therapist guidance in structuring and navigating through the process of activation, collaborative stance, and use of homework assignments were valued by participants from BA intervention program (Table 6) and echoed by those in YAG (Table 7).

In addition to facilitating aspects of BA, participant in these studies identified some barriers and challenges to practice of BA. These included sustaining behavior changes over time, as assigned activities were perceived as strenuous and not to their liking; and a strong emphasis on individual’s actions, especially in the psychoeducation component that made a few young people feel that they were individually responsible for ‘fixing’ themselves (Table 6). Both these themes also emerged during YAG discussions (Table 7).

Themes from qualitative studies and YAG discussion helped identify changes that can be made to improve intervention acceptability. These included using simple language during sessions and in the materials designed for young people; staggering out sessions at the end to sustain the learned techniques; utilization of supportive social networks and connections with peers who had similar lived experiences of mental health; personal acceptance of one’s mental health and its management; including activities that are meaningful or important to a person rather than focusing on the prescriptive set of pleasurable activities; and disseminating information about BA in proper manner to avoid misconception around “quick-fix” and “individual’s responsibility” (Table 6, 7).

During workshops, youth advisors highlighted various domains for future research in LMICs like India. These included the following (not in any particular order of priority): (i) examining the impact and limitations of BA across the range of depression and anxiety disorders; (ii) developing programs to implement and sustain BA interventions in educational settings; (iii) understanding the role of social-contextual factors in the effectiveness of BA; (iv) understanding factors that influence sustained behavioral changes among individuals following participation in the BA program; (iv) effectiveness of BA when delivered in different formats such as individual, groups, and digitally; (v) effectiveness of BA for varying severity and chronicity of problems; (vi) more research and implementation work in LMICs context; (vii) greater involvement of youth in planning interventions and their dissemination.

## Discussion

The study aimed to systematically review quantitative and qualitative studies and synthesize evidence on BA as an active intervention ingredient for addressing depression and anxiety among young people. The synthesis of evidence indicated there have not been sufficient number of good quality studies to establish the true potency of BA. The findings from a limited number of studies have indicated promising outcomes for BA in depression. Standalone BA intervention produced a superior intervention effect when compared to the inactive control condition, and comparisons to active controls showed non-significant differences between BA and other complex psychological treatments. BA has been frequently used as part of multicomponent interventions, which, excluding the cognitive elements, were similar in composition to standalone BA. These interventions similarly showed favorable outcomes, particularly for subthreshold depression (greater number of superior or valid outcomes for indicated interventions than treatment interventions). However, BA’s role as an active intervention ingredient was difficult to establish in these interventions in the absence of focal assessment of activation in most studies. There was more robust evidence for the acceptability of BA in accounts by young people with lived experience of depression. Young people appreciated BA intervention programs for impetus on actions that helped improve mood and functioning across domains, notwithstanding certain concerns they had about sustaining behavioral changes and emphasizing individuals’ responsibility for change. Young people frequently reported using behavioral strategies similar to BA in their habitual coping, while cognitive strategies were least frequently reported, which further helped strengthen the evidence on the acceptability of BA in this age group. Overall, these findings, in line with the previous reviews [16, 17, 57], indicate the potential role of BA as an active intervention ingredient for depression among young people.

The current review also examined the characteristics of BA intervention protocols. Findings indicated that BA has been typically used as part of interventions that are fairly complex, comprising multiple behavioral elements that have been used for increasing reinforcements for positive behavior and overcoming challenges towards targeted changes; however, avoidance processes underlying depression have been targeted only in a few interventions. While activity scheduling, monitoring and goal setting have been most frequently used in the BA interventions, evidence synthesis indicated that two skill-training elements, i.e., problem-solving skills and social networks and support, may play an important role in enhancing the impact of BA for this age group. Problem-solving skills have been most frequently included along with BA in both standalone and multicomponent interventions to facilitate meeting individualized goals and adopting a positive stance to overcoming barriers [e.g., 33,34,45]. Unlike problem-solving skills, social networks and support have been given limited attention in existing quantitative studies of BA [e.g., 41,44]. However, insights from qualitative studies and YAG discussions have strongly indicated the importance of strengthening this element in BA interventions, as sustained behavioral change may be limited where the wider social networks are not taken into account. These are preliminary suggestions based on limited data available about intervention composition. However, as more is learned about behavioral difficulties and processes that underlie depression (such as increasing activation, reducing avoidance, reward processing), it will become increasingly possible to measure the impact of various elements on these processes, which in turn will guide the development of a comprehensive and efficient intervention structure.

We found limited use of standardized tools to assess activation and other BA related skills in this age group. Self-monitoring, on the other hand, was frequently used to examine engagement with activities and their impact on mood. This opens up a debate on the extent to which a standardized tool like Behavioral Activation for Depression Scale (BADS) [58] versus an idiographic measure or ecological momentary assessment derived from self-monitoring can be used effectively in assessing activation. Existing literature suggests that idiographic measures are typically more sensitive to change than the standardized measures of skills assessment and more reflective of youths’ “voices” in identifying and addressing health concerns [59–61]. Further, technology aids such as mobile app and wearable sensor devices can facilitate ecological momentary assessments and provide easily accessible feedback for youth and providers [62, 63]. Systematic research will be needed on how these measures can be structured throughout the intervention to assess, monitor, and evaluate activation, which in turn, helps build evidence for the role of BA as an ‘active ingredient’ in youth interventions.

The current review, similar to previous systematic reviews [16], has found limited evidence on use and acceptability BA on anxiety. However, the focus on avoidance and graded behavioral hierarchies in the recent adaptations of BA [11, 64, 65], makes it particularly appealing as a transdiagnostic intervention, for both anxiety and depression. Future research needs to examine how the progressive structure of BA can be optimized for heterogenous anxiety problems (generalized anxiety, social anxiety, phobias) that differ in presentation and degree of overlap with depression.

A number of methodological limitations need to be considered when interpreting the findings of the current study. First, there was considerable heterogeneity in the nature of studies and outcomes, which needs to be considered when interpreting the findings from the current review. Second, there was an underrepresentation of young adults (than adolescents), males (than females), and individuals from LMICs (than HICs) and this may limit generalizability to the whole age group of adolescents and young adults across cultures. Third, many of the included studies were published before reporting guidelines (such as PRISMA) existed. Thus, the risk of bias in many studies was high, indicating need for well-powered, high-quality studies. Fourth, the main PWEBS database focused on samples aged under 21 years and we extracted data on older samples using a supplementary transition age youth database without validated codes. To improve reliability of these codes, two project researchers independently coded these studies and there was good inter-rater reliability for all categories. Fifth, there was the paucity of acceptability studies of BA among young people. We extrapolated findings from studies on coping strategies to offer implications for BA; however, more qualitative studies are needed to understand acceptability concerns from youths’ perspective. Lastly, the involvement of YAG in this review was only consultative. The usage of participatory research models and collaborative data analysis can support more meaningful involvement of YAG’s as co-analysts in future projects.

### Directions for future research

The results of the current review provide preliminary evidence and should promote further research on uncovering acceptability, adaptations and effectiveness of BA interventions for depression and anxiety through high-quality randomized control trials and mixed-methods studies. Majority of the existing evidence comes from studies conducted in high-income countries, with females and younger populations (in age range of 14–19 years). It is important to explore how this intervention could be applied in low-resource settings, with males and gender minorities, and young adults, given that specifics of and access to positively reinforcing activities may vary across settings and demographics. Both standalone and multicomponent BA interventions reviewed in this study consisted of multiple behavioral elements arranged in varying sequences across treatment protocols. Conducting dismantling studies will be important for identifying active intervention ingredients in these complex intervention protocols. Dismantling studies (e.g., using Interrupted Time Series and Sequential Multiple Assignment Randomized Trial designs) would enable the comparison of discrete BA elements with complex, multiple component protocols to identify the degree to which specific components add to the effectiveness of intervention and the distinctive mechanisms of change underlying their effectiveness for depression, anxiety or both. Based on current review, some of the clinically relevant topics to be investigated are: benefits of personalizing activity selection and scheduling in implementation of BA, sustaining behavioral changes over time, effectiveness of formats and modalities (individual versus group, face-to-face versus digital); and delivery of BA by non-specialists. There is preliminary evidence from both young people and adult literature that BA delivered through lay-counsellors can be effective and address the supply side of the demand–supply gap in accessing mental health care [6, 7, 47]. Further, as studies indicated BA has relevance beyond formalized therapeutic settings and can be extended to natural environment for facilitating coping with mental health problems. Thus, setting up youth activity clubs based on BA principles is an important area that needs further exploration. These research priorities are not only relevant from a clinical and research perspective but are also in line with YAG's priorities. There may be value in soliciting youth perspectives on what might be effective based on their own life experiences, which may help generate hypotheses about ways to streamline and strengthen interventions that are designed to target their mental health and well-being.

## Conclusions

In line with previous reviews [16, 17], the current rapid review found preliminary evidence for the effectiveness of BA in the treatment of depression, with no evidence for its use in anxiety among young people. There was more robust evidence in accounts of young people for BA as an acceptable coping strategy for improving mood and activity levels. Overall, more research is needed to examine the components and mechanisms that contribute to BA's effectiveness as an active intervention ingredient for depression and anxiety.

## Supplementary Information

**Additional file 1.** Demographics and lived experience of depression and anxiety among members of project’s Youth Advisory Group (YAG).

**Additional file 2.** Outcome measures for depression, anxiety, functioning and activation used in RCT studies.

**Additional file 3.** References of all studies included in the review.

**Additional file 4.** Additional files legend.

## Data Availability

All data generated or analysed during this study are included in this manuscript and the additional files.

## Abbreviations

BA: Behavioral activation
BADS: Behavioral activation for depression scale
HICs: High-income countries
LMICs: Low-middle income countries
MMAT: Mixed methods appraisal tool
PWEBS: PracticeWise evidence-based services
RCT: Randomized control trials
RoB: Risk of bias
YAG: Youth advisory group
