# Supplementary material for: Behavioral Activation as an ‘active ingredient’ of interventions addressing depression and anxiety among young people: a systematic review and evidence synthesis
Source: BMC Psychol. 2021 Oct 7;9:150. doi: 10.1186/s40359-021-00655-x (PMC8494510; doi:10.1186/s40359-021-00655-x)
Supplement: Supplementary file 1 — Additional file 1. Demographics and lived experience of depression and anxiety among members of project’s Youth Advisory Group (YAG). [file 40359_2021_655_MOESM1_ESM.docx]

**Additional file 1**

**Behavioral Activation as an ‘active ingredient’ of interventions addressing depression and anxiety among young people: a systematic review and evidence synthesis**

Kanika Malik, Maliha Ibrahim, Adam Bernstein, Rahul KV, Tara Rai, Bruce Chorpita and Vikram Patel

**Table S1: Demographics and lived experience of depression and anxiety among members of project’s Youth Advisory Group**

| **YAG members id** | **Age** | **Gender** | **Nationality** | **Lived experience of depression (self-reported)** | **Lived experience of anxiety (self-reported)** | **Received psychosocial intervention (self-reported)** |
| --- | --- | --- | --- | --- | --- | --- |
| Member 1 | 15 | Male | Indian | Yes | Yes | Yes |
| Member 2 | 16 | Female | Indian | No | Yes | Yes |
| Member 3 | 17 | Female | Indian | Yes | Yes | No |
| Member 4 | 19 | Non-binary | Indian | Yes | No | No |
| Member 5 | 20 | Female | Indian | Yes | Yes | Yes |
| Member 6 | 20 | Female | Indian | Yes | Yes | Yes |
| Member 7 | 21 | Male | Indian | Yes | Yes | Yes |
| Member 8 | 23 | Female | Indian | Yes | Yes | Yes |
| Member 9 | 24 | Transman | Indian | No | Yes | Yes |
| Member 10 | 21 | Female | Indian | Yes | Yes | Yes |
