# Supplementary material for: Behavioral Activation as an ‘active ingredient’ of interventions addressing depression and anxiety among young people: a systematic review and evidence synthesis
Source: BMC Psychol. 2021 Oct 7;9:150. doi: 10.1186/s40359-021-00655-x (PMC8494510; doi:10.1186/s40359-021-00655-x)
Supplement: Supplementary file 2 — Additional file 2. Outcome measures for depression, anxiety, functioning and activation used in RCT studies. [file 40359_2021_655_MOESM2_ESM.docx]

**Additional file 2**

**Behavioral Activation as an ‘active ingredient’ of interventions addressing depression and anxiety among young people: a systematic review and evidence synthesis**

Kanika Malik, Maliha Ibrahim, Adam Bernstein, Rahul KV, Tara Rai, Bruce Chorpita and Vikram Patel

**Table S2: Outcome measures for depression, anxiety, functioning and activation used in RCT studies^*^**

|  | **Studies with BA as standalone intervention (n=3)** | **Studies with BA as element in multicomponent intervention (n=20)** |
| --- | --- | --- |
| Depression | Mood and Feeling Questionnaire (n=1), Children's Depression Rating Scale – Revised (n=1), Beck Depression Inventory II - Japanese Version (n=1) | Beck Depression Inventory II (n=6), Beck Depression Inventory (1961) (n=4), Beck Depression Inventory - 13 Item Short Version (n=1), Children's Depression Inventory (n=2), Children's Depression Rating Scale – Revised (n=2), Quick Inventory of Depressive Symptomatology- Adolescent (2007) (n=1), Center for Epidemiologic Studies Depression Scale - Chinese Version (n=1), Patient Health Questionnaire-9 (n=1), Center for Epidemiologic Studies Depression Scale (n=2) |
| Anxiety | The Multidimensional Anxiety Scale for Children (n=1), Revised Children’s Manifest Anxiety Scale (n=1) | Depression, Anxiety, Stress Scale (n=1), State Anxiety Questionnaire (n=1), Beck Anxiety Inventory (n=1), Anxiety subscale of the Hospital Anxiety and Depression Scale (n=1), Spence Children’s Anxiety Scale (n=1) |
| Functioning | Health Of The Nation Outcome Scales For Children And Adolescents (n=1), Children's Global Assessment Scale (n=1), EuroQOL - 5 Dimension - Japanese Version (n=1) | Children's Global Assessment Scale (n=1), Global Assessment of Functioning Scale (n=1), Clinician Global Ratings of Improvement and Severity (n=1), Social Adjustment Scale - Self-Report for Youth (n=1), Children's Global Assessment Scale (n=2), Social Adjustment Scale for Children and Adolescents (SASCA) (n=1), Paediatric Quality of Life Inventory (n=1) |
| Activation | Behavioral Activation for Depression Scale (n=1), The Japanese version of the Behavioral Activation for Depression Scale (n=1) | NA |
| *For each intervention, we included only one outcome measure for each domain, based on PracticeWise Evidence-Based Services (PWEBS) coding of the primary measure for that domain | | |
