# Supplementary material for: Behavioral Activation as an ‘active ingredient’ of interventions addressing depression and anxiety among young people: a systematic review and evidence synthesis
Source: BMC Psychol. 2021 Oct 7;9:150. doi: 10.1186/s40359-021-00655-x (PMC8494510; doi:10.1186/s40359-021-00655-x)
Supplement: Supplementary file 3 — Additional file 3. References of all studies included in the review. [file 40359_2021_655_MOESM3_ESM.docx]

**Additional file 3**

**Behavioral Activation as an ‘active ingredient’ of interventions addressing depression and anxiety among young people: a systematic review and evidence synthesis**

Kanika Malik, Maliha Ibrahim, Adam Bernstein, Rahul KV, Tara Rai, Bruce Chorpita and Vikram Patel

**References of all studies included in the review**

1. Goodyer IM, Reynolds S, Barrett B, Byford S, Dubicka B, Hill J, et al. Cognitive-behavioural therapy and short-term psychoanalytic psychotherapy versus brief psychosocial intervention in adolescents with unipolar major depression (IMPACT): a multicentre, pragmatic, observer-blind, randomised controlled trial. Health Technol Assess. 2017 Mar;21(12):1–94..
2. McCauley E, Gudmundsen G, Schloredt K, Martell C, Rhew I, Hubley S, et al. The Adolescent Behavioral Activation Program: Adapting Behavioral Activation as a Treatment for Depression in Adolescence. J Clin Child Adolesc Psychol. 2016;45(3):291–304.
3. Takagaki K, Okamoto Y, Jinnin R, Mori A, Nishiyama Y, Yamamura T, Yokoyama S, Shiota S, Okamoto Y, Miyake Y, Ogata A. Behavioral activation for late adolescents with subthreshold depression: a randomized controlled trial. Eur Child Adolesc Psychiatry. 2016 Nov 1;25(11):1171-82.
4. Brent DA, Holder D, Kolko D, Birmaher B, Baugher M, Roth C, Iyengar S, Johnson BA. A clinical psychotherapy trial for adolescent depression comparing cognitive, family, and supportive therapy. Arch Gen Psychiatry. 1997 Sep 1;54(9):877-85.
5. Burton E, Stice E, Bearman SK, Rohde P. Experimental test of the affect‐regulation theory of bulimic symptoms and substance use: A randomized trial. Int J Eat Disord. 2007 Jan;40(1):27-36.
6. Clarke GN, Rohde P, Lewinsohn PM, Hops H, Seeley JR. Cognitive-behavioral treatment of adolescent depression: efficacy of acute group treatment and booster sessions. J Am Acad Child Adolesc Psychiatry. 1999 Mar 1;38(3):272-9.
7. Deady M, Mills KL, Teesson M, Kay-Lambkin F. An Online Intervention for Co-Occurring Depression and Problematic Alcohol Use in Young People: Primary Outcomes From a Randomized Controlled Trial. J Med Internet Res [Internet]. 2016 Mar 23 [cited 2020 Sep 7];18(3). Available from: https://www.ncbi.nlm.nih.gov/pmc/articles/PMC4823588/
8. Ip P, Chim D, Chan KL, Li TM, Ho FK, Van Voorhees BW, Tiwari A, Tsang A, Chan CW, Ho M, Tso W. Effectiveness of a culturally attuned Internet‐based depression prevention program for Chinese adolescents: A randomized controlled trial. Depress Anxiety. 2016 Dec;33(12):1123-31.
9. Kobak KA, Mundt JC, Kennard B. Integrating technology into cognitive behavior therapy for adolescent depression: a pilot study. Ann Gen Psychiatry. 2015 Dec 1;14(1):37.
10. Lewinsohn PM, Clarke GN, Hops H, Andrews J. Cognitive-behavioral treatment for depressed adolescents. Behavior Therapy. 1990 Sep 1;21(4):385-401.
11. Ranney ML, Pittman SK, Dunsiger S, Guthrie KM, Spirito A, Boyer EW, Cunningham RM. Emergency department text messaging for adolescent violence and depression prevention: A pilot randomized controlled trial. Psychol Serv.. 2018 Nov;15(4):419.
12. Reynolds WM, Coats KI. A comparison of cognitive-behavioral therapy and relaxation training for the treatment of depression in adolescents. J Consult Clin Psychol. 1986 Oct;54(5):653–60.
13. Rohde P, Clarke GN, Mace DE, Jorgensen JS, Seeley JR. An efficacy/effectiveness study of cognitive-behavioral treatment for adolescents with comorbid major depression and conduct disorder. J Am Acad Child Adolesc Psychiatry. 2004 Jun;43(6):660–8.
14. Rosselló J, Bernal G. The efficacy of cognitive-behavioral and interpersonal treatments for depression in Puerto Rican adolescents. J Consult Clin Psychol. 1999 Oct;67(5):734.
15. Rosselló J, Bernal G, Rivera-Medina C. Individual and group CBT and IPT for Puerto Rican adolescents with depressive symptoms. Cultur Divers Ethnic Minor Psychol. 2008 Jul;14(3):234.
16. Shirk SR, DePrince AP, Crisostomo PS, Labus J. Cognitive behavioral therapy for depressed adolescents exposed to interpersonal trauma: An initial effectiveness trial. Psychotherapy (Chic). 2014 Mar;51(1):167-79
17. Stasiak K, Hatcher S, Frampton C, Merry SN. A pilot double blind randomized placebo controlled trial of a prototype computer-based cognitive behavioural therapy program for adolescents with symptoms of depression. Behav Cogn Psychother. 2014;42(4):385–401.
18. Stice E, Burton E, Bearman SK, Rohde P. Randomized Trial of a Brief Depression Prevention Program. Behav Res Ther. 2007 May;45(5):863–76.
19. March J, Silva S, Petrycki S, Curry J, Wells K, Fairbank J, Burns B, Domino M, McNulty S, Vitiello B, Severe J; Treatment for Adolescents With Depression Study (TADS) Team. Fluoxetine, cognitive-behavioral therapy, and their combination for adolescents with depression: Treatment for Adolescents With Depression Study (TADS) randomized controlled trial. JAMA. 2004 Aug 18;292(7):807-20.
20. Tandon SD, Perry DF, Mendelson T, Kemp K, Leis JA. Preventing perinatal depression in low-income home visiting clients: a randomized controlled trial. J Consult Clin Psychol. 2011 Oct;79(5):707-12.
21. Topooco N, Berg M, Johansson S, Liljethörn L, Radvogin E, Vlaescu G, et al. Chat- and internet-based cognitive-behavioural therapy in treatment of adolescent depression: randomised controlled trial. BJPsych Open. 2018;4(4):199–207.
22. van der Zanden R, Kramer J, Gerrits R, Cuijpers P. Effectiveness of an online group course for depression in adolescents and young adults: a randomized trial. J Med Internet Res. 2012 Jun 7;14(3):e86.
23. Wright B, Tindall L, Littlewood E, Allgar V, Abeles P, Trépel D, Ali S. Computerised cognitive–behavioural therapy for depression in adolescents: feasibility results and 4-month outcomes of a UK randomised controlled trial. BMJ open. 2017 Jan 1;7(1):e012834.
24. Arnott B, Kitchen CE, Ekers D, Gega L, Tiffin PA. Behavioural activation for overweight and obese adolescents with low mood delivered in a community setting: feasibility study. BMJ Paediatrics Open. 2020;4(1).
25. Bru L, Solholm R, Idsoe T. Participants’ experiences of an early cognitive behavioral intervention for adolescents with symptoms of depression. Emot Behav Diffic. 2013;18(1):24–43.
26. Iloabachie C, Wells C, Goodwin B, Baldwin M, Vanderplough-Booth K, Gladstone T, Murray M, Fogel J, Van Voorhees BW. Adolescent and parent experiences with a primary care/Internet-based depression prevention intervention (CATCH-IT). General Hospital Psychiatry. 2011 Nov 1;33(6):543-55.
27. Al-Khattab H, Oruche U, Perkins D, Draucker C. How African American adolescents manage depression: Being with others. J Am Psychiatr Nurses Assoc. 2016 Sep;22(5):387-400.
28. Aselton P. Sources of stress and coping in American college students who have been diagnosed with depression: Sources of stress and coping in American college students who have been diagnosed with depression. J Child Adolesc Psychiatr Nurs. 2012;25(3):119–23.
29. Bluhm RL, Covin R, Chow M, Wrath A, Osuch EA. “I just have to stick with it and it’ll work”: experiences of adolescents and young adults with mental health concerns. Community Ment Health J.. 2014 Oct 1;50(7):778-86.
30. Boyd C, Hayes L, Nurse S, Aisbett D, Francis K, Newnham K, et al. Preferences and intention of rural adolescents toward seeking help for mental health problems. Rural Remote Health [Internet]. 2011 [cited 2020 Sep 6];11, no. 1 (2011), pp. 1–13. Available from: <https://researchonline.federation.edu.au/vital/access/manager/Repository/vital:5243>
31. Breland-Noble AM, Burriss A, Poole HK, AAKOMA PROJECT Adult Advisory Board. Engaging depressed African American adolescents in treatment: lessons from the AAKOMA PROJECT. J Clin Psychol. 2010;66(8):868–79.
32. Breland-Noble AM, Wong MJ, Childers T, Hankerson S, Sotomayor J. Spirituality and religious coping in African American youth with depressive illness. Ment Health Relig Cult. 2015;18(5):330–41.
33. Chernomas WM, Shapiro C. Stress, depression, and anxiety among undergraduate nursing students. Int J Nurs Educ Scholarsh. 2013;10(1):255–66.
34. Dundon EE. Adolescent depression: a metasynthesis. J Pediatr Health Care. 2006 Nov-Dec;20(6):384-92..
35. Farmer TJ. The experience of major depression: adolescents' perspectives. Issues Ment Health Nurs. 2002 Sep;23(6):567-85.
36. Fornos LB, Seguin Mika V, Bayles B, Serrano AC, Jimenez RL, Villarreal R. A qualitative study of Mexican American adolescents and depression. J Sch Health. 2005 May;75(5):162-70.
37. Grob R, Schlesinger M, Wise M, Pandhi N. Stumbling into adulthood: Learning from depression while growing up. Qual Health Res. 2020;30(9):1392–408.
38. Hannor-Walker TL. Overlooked and undertreated: A qualitative study of African American adolescents and depression in southwest Georgia [dissertation on the internet]. Georgia: Capella University; 2008. Accessed from: https://www.proquest.com/openview/e2883490ad6d8eb1a39a9e9be4ac3de3/1?pq-origsite=gscholar&cbl=18750
39. Kuwabara SA, Van Voorhees BW, Gollan JK, Alexander GC. A qualitative exploration of depression in emerging adulthood: disorder, development, and social context. Gen Hosp Psychiatry. 2007 Jul 1;29(4):317-24.
40. Martínez-Hernáez A, Carceller-Maicas N, DiGiacomo SM, Ariste S. Social support and gender differences in coping with depression among emerging adults: a mixed-methods study. Child Adolesc Psychiatry Ment Health. 2016 Jan 7;10:2.
41. Martínez-Hernáez A, DiGiacomo SM, Carceller-Maicas N, Correa-Urquiza M, Martorell-Poveda MA. Non-professional-help-seeking among young people with depression: a qualitative study. BMC psychiatry. 2014 Dec;14(1):1-1.
42. McCarthy J, Downes E, Sherman C. Looking back at adolescent depression: A qualitative study. J Ment Health Couns. 2008;30(1):49–68.
43. McCann TV, Lubman DI, Clark E. Views of young people with depression about family and significant other support: Interpretative phenomenological analysis study: support and young people with depression. Int J Ment Health Nurs. 2012;21(5):453–61.
44. Morey-Nase C, Phillips LJ, Bryce S, Hetrick S, Wright AL, Caruana E, et al. Subjective experiences of neurocognitive functioning in young people with major depression. BMC Psychiatry. 2019 Jul 4;19(1):209.
45. Moses T. Self-labeling and its effects among adolescents diagnosed with mental disorders. Soc Sci Med. 2009;68(3):570–8.
46. Ofonedu ME, Percy WH, Harris-Britt A, Belcher HME. Depression in inner city African American youth: A phenomenological study. J Child Fam Stud. 2013;22(1):96–106.
47. Oliver J, Smith P, Leigh E. ‘All these negative thoughts come flooding in’: how young people with depression describe their experience of rumination. Cogn Behav Therap [Internet]. 2015;8(e15). Available from: http://dx.doi.org/10.1017/s1754470x15000306
48. Özkul B, Günüşen NP. Stressors and Coping Methods of Turkish Adolescents With High and Low Risk of Depression: A Qualitative Study. J Am Psychiatr Nurses Assoc. 2020 Jan 22:1078390319895088.
49. Recto P, Champion JD. “We don't want to be judged”: perceptions about professional help and attitudes towards help-seeking among pregnant and postpartum Mexican-American adolescents. J Pediatr Nurs. 2018 Sep 1;42:111-7.
50. Ross E, Ali A, Toner B. Investigating issues surrounding depression in adolescent girls across Ontario: A participatory action research project. Can J Commun Ment Health. 2003;22(1):55–68.
51. Ross VM. More than “a little bit nervous”: understanding the experiences of young women with anxiety during secondary school [master's thesis on the internet]. Canada: Queen’s University; 2015. Accessed from: https://qspace.library.queensu.ca/bitstream/handle/1974/13764/Ross_Vita-Marie_201509_MED.pdf?sequence=1
52. Sabiston CM, Sedgwick WA, Crocker PRE, Kowalski KC, Mack DE. Social physique anxiety in adolescence: An exploration of influences, coping strategies, and health behaviors. J Adolesc Res. 2007;22(1):78–101.
53. Sam P. Young Adults Attending College Who Experience Depression: A Qualitative Study [dissertation on the internet]. Virginia: School of Psychology & Counseling Regent University; 2019
54. Simonds LM, Pons RA, Stone NJ, Warren F, John M. Adolescents with anxiety and depression: Is social recovery relevant?: Adolescents with anxiety and depression. Clin Psychol Psychother. 2014;21(4):289–98.
55. Weitkamp K, Klein E, Midgley N. The experience of depression: A qualitative study of adolescents with depression entering psychotherapy. Glob Qual Nurs Res. 2016;3:233339361664954.
56. Wisdom JP, Green CA. “being in a funk”: Teens’ efforts to understand their depressive experiences. Qual Health Res. 2004;14(9):1227–38.
57. Wisdom JP, Agnor C. Family heritage and depression guides: Family and peer views influence adolescent attitudes about depression. J Adolesc. 2007;30(2):333–46.
58. Wisdom JP, Barker EC. Getting out of depression: Teens’ self-help interventions to relieve depressive symptoms. Child Fam Behav Ther. 2006;28(4):1–11.
59. Woodgate RL. Living in the shadow of fear: adolescents’ lived experience of depression. J Adv Nurs. 2006;56(3):261–9.
60. Woodgate RL, Tailor K, Tennent P, Wener P, Altman G. The experience of the self in Canadian youth living with anxiety: A qualitative study. PLoS One. 2020;15(1):e0228193.
