# Supplementary material for: Behavioral Activation as an ‘active ingredient’ of interventions addressing depression and anxiety among young people: a systematic review and evidence synthesis
Source: BMC Psychol. 2021 Oct 7;9:150. doi: 10.1186/s40359-021-00655-x (PMC8494510; doi:10.1186/s40359-021-00655-x)
Supplement: Supplementary file 4 — Additional file 4. Additional files legend. [file 40359_2021_655_MOESM4_ESM.docx]

**Additional files legend**

Additional file 1.docx

Demographics and lived experience of depression and anxiety among members of project’s Youth Advisory Group

Additional file 2.docx

Outcome measures for depression, anxiety, functioning and activation used in RCT studies

Additional file 3.docx

References of all studies included in the review
